# Anti-N-methyl-D-aspartate receptor encephalitis after coronavirus disease 2019: A case report and literature review

**DOI:** 10.1097/MD.0000000000030464

**Published:** 2022-09-02

**Authors:** Hyesun Lee, Jong Hyun Jeon, Hojin Choi, Seong-Ho Koh, Kyu-Yong Lee, Young Joo Lee, Hyuk Sung Kwon

**Affiliations:** a Department of Neurology, Hanyang University College of Medicine, Hanyang University Guri Hospital, Guri, Republic of Korea.

**Keywords:** anti-N-methyl-D-aspartate receptor encephalitis, case report, COVID-19, ovarian teratoma

## Abstract

Coronavirus disease 2019 (COVID-19) has become a global pandemic and COVID-19-associated anti-N-methyl-D-aspartate receptor (NMDAR) encephalitis may occur through an immune-mediated pathomechanism.

**Patient concerns:**

A 21-year-old woman with a history of COVID-19 presented to our hospital with memory decline and psychiatric symptoms.

**Diagnosis:**

The patient was diagnosed with anti-NMDAR encephalitis.

**Intervention:**

Intravenous methylprednisolone (1 g/day over 5 days) followed by immunoglobulin (0.4 g/kg/day over 5 days) were administered. The patient underwent laparoscopic salpingo-oophorectomy to remove an ovarian teratoma.

**Outcomes:**

The patient was discharged with sequelae of short-term memory impairment, without other neuropsychiatric symptoms.

**Lessons:**

Cases of previously reported anti-NMDAR encephalitis with COVID-19 were reviewed and compared with the present case. Clinicians should be aware of the occurrence of anti-NMDAR encephalitis in patients who present with neuropsychiatric complaints during or after exposure to COVID-19. Further studies are required to determine the causal relationship between the 2 diseases and predict the prognosis of anti-NMDAR encephalitis after COVID-19 exposure.

## 1. Introduction

Anti-N-methyl-D-aspartate receptor (NMDAR) encephalitis is the most common form of autoimmune encephalitis, occurring more frequently in young women. It is also associated with tumors, especially ovarian teratomas.^[1]^ About 80% of patients with anti-NMDAR encephalitis benefit from adaptive immunotherapy with or without removal of teratomas, and early tumor removal is associated with good prognosis.^[1–4]^

Coronavirus disease 2019 (COVID-19) pandemic, caused by the severe acute respiratory syndrome coronavirus 2 (SARS-CoV-2), occurred in 2019, and various neurological diseases associated with it have been continuously reported. SARS-CoV-2 can invade the central nervous system through systemic circulation via angiotensin-converting enzyme 2 receptors and the cribriform plate.^[5]^

Until now, only a few cases of anti-NMDAR encephalitis associated with COVID-19 have been reported.^[6,7]^ Structural similarities between NMDAR and a subunit of SARS-CoV-2 may trigger anti-NMDAR encephalitis after COVID-19.^[6]^ We report a case of a patient with anti-NMDAR encephalitis triggered by COVID-19. To the best of our knowledge, this is the first such case reported in East Asia.

## 2. Case report

A 21-year-old woman visited the emergency department with a complaint of short-term memory loss and abnormal behavior for past 1 week. She repeated the same words and presented an incoherent speech. She had no known underlying diseases and received the third dose of BNT162b2 vaccination against SARS-CoV-2 4 months prior to the visit. A polymerase chain reaction test performed on the nasopharyngeal swab collected from her approximately 10 days prior to admission was positive for COVID-19. Her abnormal behavior was noticed 3 days after SARS-CoV-2 infection was detected.

**Figure 1.**
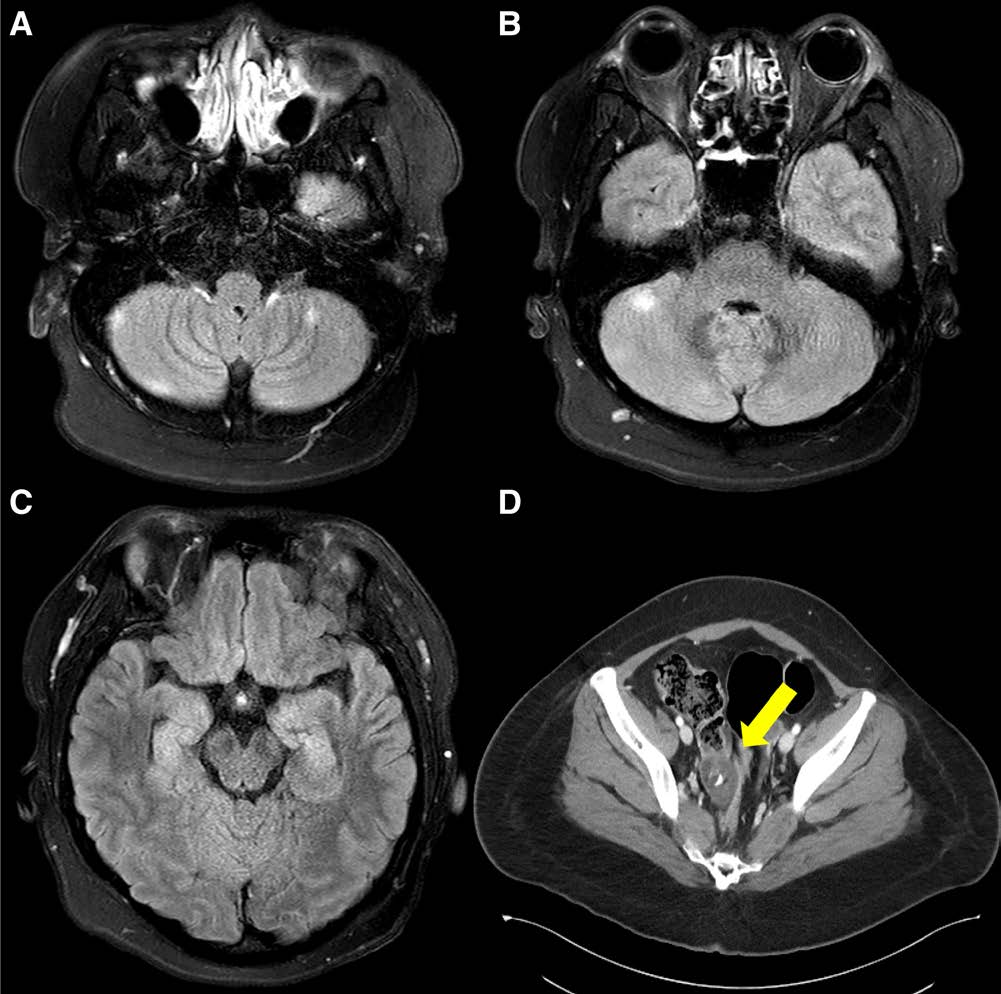
Brain magnetic resonance images (A–C) and enhanced abdominopelvic computed tomography (CT) image of the patient (D). Enhanced fluid-attenuated inversion recovery (FLAIR) images show enhancing lesions in bilateral cerebellum (A, B) and hippocampus (C). CT shows right ovarian mass with calcification suggestive of teratoma (D, arrow).

Laboratory tests revealed no abnormalities. Cerebrospinal fluid (CSF) analysis revealed a high opening pressure of 252 mm H_2_O, white blood cell count of 500/mm^3^ (90% lymphocytes), red blood cell count of 85,700/mm^3^, protein level of 402.4 mg/dL, glucose level of 57.8 mg/dL (serum glucose level of 104 mg/dL), adenosine deaminase level of 5.9 IU/L, and corrected white blood cell count of 642.92/mm^3^. Brain fluid-attenuated inversion recovery images showed contrast-enhanced lesions in the cerebellum and hippocampus (Fig. 1A-C). Electroencephalography exhibited diffuse beta wave activity with rare sharp waves in both the temporal lobes. Chest and abdomen-pelvic computed tomography revealed a mass of 5 cm in size in the right ovary, suspected to be a teratoma (Fig. 1D). Subsequently, her serum and CSF specimens were found to be positive for anti-NMDAR antibodies. The CSF oligoclonal band was negative; moreover, the serum was negative for paraneoplastic autoantibodies such as anti-Hu, Ri, Yo, amphiphysin, CV2, PNMA2 (Ma2/Ta), Recoverin, SOX1, and Titin.

Intravenous acyclovir (10 mg/kg per 8 hours for 8 days) and corticosteroids (methylprednisolone 1 g/day for 5 days) were initiated. Laparoscopic right salpingo-oophorectomy was performed. The psychosis, anxiety, and memory loss persisted even after treatment with corticosteroids. Therefore, intravenous immunoglobulin (0.4 g/kg/day over 5 days) was administered. She was discharged with improvement in psychosis. However, recovery of her memory impairment was incomplete, which would require continuous monitoring.

## 3. Discussion

Anti-NMDAR encephalitis occurs when antibodies against NMDAR are produced, and is triggered by herpes simplex type 1 encephalitis and tumors, including ovarian teratomas.^[1]^ However, infection with diverse viruses including the Japanese encephalitis virus could also elicit anti-NMDAR encephalitis.^[1,8]^ Furthermore, H1N1, polio, tetanus, diphtheria, and pertussis vaccination is also related to the manifestation of anti-NMDAR encephalitis.^[8]^

Recently with the occurrence of the COVID-19 pandemic, some research regarding the relationship between COVID-19 and anti-NMDAR encephalitis has emerged. The subunits of NMDAR and non-structural proteins 8 and 9 in SARS-CoV-2 are structurally similar, and this mimicry may affect the cross-reactivity between them.^[6]^ Furthermore, COVID-19 increases the release of inflammatory markers from the alveolar epithelium and macrophages. This leads to increased vascular permeability, and disruption of the blood-brain barrier.^[9]^ Blood-brain barrier breakdown raises the risk of NMDAR antibodies invading the central nervous system.^[6,10]^

Among more than 50 micro-ribonucleic acid biomarkers of COVID-19, 7 are known to be related to anti-NMDAR encephalitis.^[4]^ These common biomarkers are miR-107, miR-29b, let-7a, let-7f, miR-26b, miR-21, and miR-155; they do not contain the main biomarker for anti-NMDAR encephalitis (let-7b), yielding a ratio of <0.2. This may explain the low risk of anti-NMDAR encephalitis occurrence after COVID-19 infection. However, theses common micro-ribonucleic acid biomarkers may explain the causal relationship between COVID-19 and anti-NMDAR encephalitis. COVID-19 might trigger anti-NMDAR encephalitis using these common biomarkers, but the risk of occurrence may be low.^[4]^

We reviewed published case reports of anti-NMDAR encephalitis related to COVID-19 in adults (aged ≥18 years or more) and found a total of 5 adult patients with anti-NMDAR encephalitis associated with COVID-19 (Table 1).^[11–15]^ All these patients had psychiatric or behavioral symptoms and received immunotherapies such as steroids and intravenous immunoglobulin. However, unlike the previous 5 cases, our case had some distinct features.

**Table 1:** Literature review of cases of anti-N-methyl-D-aspartate receptor encephalitis associated with Coronavirus disease 2019.

No	Article	Age/sex/past history	Neuropsychiatric symptoms	Results (CSF/brain imaging/EEG/sample day for the positive COVID-19, before or after the admission)	Immunotherapy/teratoma removal Surgery	Outcome
1	Bravo et al 2020^[11]^	30/F/ none	3 days prior and admission day: Psychomotor agitation, paranoid ideation, dysarthria with	CSF: Lymphocytic pleocytosis, elevated protein levels, SARS-CoV-2 PCR (-)	IVMP, IVIG, Rituximab	Improvement but cognitive sequelae
			dysprosody, and visual hallucinations	Brain MRI: Hyperintensity in Left hippocampus	Left ovarian teratoma removal	
			During HD: Buccolingual dyskinesia, chorea-dystonic movements, blepharoclonus, and focal and generalized seizures	EEG: Epileptic discharges in the left frontotemporal region. Delta brush pattern with spike and wave discharges in anterior regions		
				COVID-19 Sample: After 3 days (Nasopharyngeal swab)		
2	Panariello et al 2020^[12]^	23/M/Drug abuse	3 days prior and admission day: Psychomotor agitation, anxiety, thought disorganization, persecutory delusions, and auditory hallucinations	CSF: Did not reveal any evidence of central nervous system infection. Increased interleukin-6, SARS-CoV-2 PCR (-)	IVMP, IVIG	Improvement
			During HD: Non-verbal, non-responsive to commands, dyskinesia, and autonomic failure	Brain CT: Unremarkable	Not mentioned	Clinical conditions are ameliorating to date
				EEG: Theta activity at 6 Hz		
				COVID-19 Sample: Admission day		
3	Monti et al 2020^[13]^	50/M/Mild hypertension	Admission day: Psychiatric symptoms including confabulations, and delirious ideas	CSF: Pleocytosis, mildly elevated protein level, Increased interleukin -6, SARS-CoV-2 PCR (-)	IVMP, IVIG, Plasma exchange	Improvement
			During HD: Focal motor seizures, orofacial dyskinesia, and refractory status epilepticus	Brain MRI: Unremarkable	No teratoma	
				EEG: Delta brush pattern. Anterior sub-continuous periodic theta activity		
				COVID-19 Sample: After approximately 8 days (throat swab)		
4	McHattie et al 2021^[14]^	53/F/Ductal breast carcinoma under remission, depression	3 weeks prior: Confusion, palilalia	CSF: Lymphocytic pleocytosis, mildly elevated protein, SARS-CoV-2 PCR (-)	IVMP, IVIG, tocilizumab	Improvement, but sequelae of left hemiparesis
			Admission day: Alert	Brain CT: Unremarkable	No teratoma	
			During HD: Severe echolalia, palilalia, high-pitched voice, echopraxia, behavioral disinhibition, mildly left sided weakness, focal seizures, prominent dysautonomia, and no hyperkinetic movement disorder	Brain MRI: Hyperintensity in Lt. amygdala, and anterior putamen & subtle signal change in Rt. Amygdala		
				EEG: Slow activity but no epileptiform discharges		
				COVID-19 Sample: After 14 days (Nasopharyngeal swab)		
5	Allahyari et al 2021^[15]^	18/F/none	3 weeks prior: Mood change, anhedonia, lack of concentration	CSF: Lymphocytic pleocytosis, elevated protein, SARS-CoV-2 PCR (+)	IVMP, IVIG	Complete recovery
			Admission day: Generalized tonic-clonic seizures	Brain CT: generalized brain edema	Not mentioned	
			During HD: Deteriorated level of consciousness, and confused state	Brain MRI: Unremarkable		
				EEG: Not mentioned		
				COVID-19 Sample: Not clearly mentioned, after 1 week immunoglobulinM(+)		

Anti-NMDAR = anti-N-methyl-D-aspartate receptor, COVID-19 = coronavirus disease 2019, CSF = cerebrospinal fluid, EEG = electroencephalogram, HD = hospital day, IL = interleukin, IVIG = intravenous immunoglobulin, IVMP = intravenous methylprednisolone, PCR = polymerase chain reaction, SARS-CoV-2 = severe acute respiratory syndrome coronavirus 2.

In the current study, COVID-19 confirmation preceded the appearance of anti-NMDAR encephalitis symptoms. In previous cases, patients were confirmed to be positive for COVID-19 after their anti-NMDAR encephalitis related symptoms had appeared. The neurological manifestations of COVID-19 are similar to those of the anti-NMDAR encephalitis. Approximately 36.4% of patients with COVID-19 showed neurologic symptoms^[16]^ making it difficult to distinguish whether the first neuropsychiatric symptoms are due to COVID-19 or anti-NMDAR encephalitis.

To the best of our knowledge, this is the first reported case of anti-NMDAR encephalitis suspected to be triggered by COVID-19, in East Asia. One recent study reviewed cases of autoimmune encephalitis in COVID-19 and suggested that their prognosis was relatively good.^[7]^ Our case had relatively good prognosis except for memory impairment, and the other 5 cases had improvement of symptoms with some minor sequelae. This is similar to the outcome of the patient in our current study; however, the cognitive decline in the current patient remained significant. Additional studies with more cases are required to evaluate the prognosis of anti-NMDAR encephalitis after COVID-19.

There are some ambiguities related to the case. It is unclear whether the patient’s neuropsychiatric symptoms were due to COVID-19 or anti-NMDAR encephalitis. In addition, it cannot be conclusively assumed that the patient’s ovarian teratoma in our case had existed ever since the COVID-19 exposure. However, it can be concluded that when a patient with COVID-19 exhibits neuropsychiatric symptoms, anti-NMDAR encephalitis should be considered as a comorbidity.

Moreover, in the present case, we did not evaluate the CSF for Interleukin-6 (IL-6) or SARS-CoV-2. In COVID-19, a cytokine storm occurs and IL-6 increases during the inflammatory phase of COVID-19. In particular, elevated IL-6 levels in the CSF lead to increased production of autoantibodies in anti-NMDAR encephalitis.^[13,17]^ Hence, CSF needs to be tested for IL-6 to clarify whether COVID-19 and anti-NMDAR encephalitis occurred during a similar duration of time by coincidence or they had a causal relationship.

To date, only a few cases of anti-NMDAR encephalitis triggered by COVID-19 have been reported. We report a case of anti-NMDAR encephalitis occurring after COVID-19 exposure in South Korea and compare the results with those of previous studies. COVID-19 may act as a trigger for the occurrence of anti-NMDAR encephalitis.

If a patient shows neuropsychiatric symptoms after COVID-19, suspecting an association with anti-NMDAR encephalitis is essential and the symptoms should not be regarded as an exclusive manifestation of COVID-19. With the accumulation of cases and data related to COVID-19 and anti-NMDAR encephalitis, we can anticipate to determine the epidemiology, establish an algorithm for effective treatments, and predict the prognosis of anti-NMDAR encephalitis following COVID-19 exposure.

## Acknowledgment

The authors express thank to the patient for generously authorizing us to share her rare case.

## Author contributions

**Conceptualization:** Hyesun Lee, Hyuk Sung Kwon.

**Data curation:** Hyesun Lee, Jong Hyun Jeon.

**Investigation:** Hojin Choi, Seong-Ho Koh, Kyu-Yong Lee, Young Joo Lee, Hyesun Lee, Hyuk Sung Kwon.

**Supervision:** Hojin Choi, Seong-Ho Koh, Kyu-Yong Lee, Young Joo Lee, Hyuk Sung Kwon.

**Visualization:** Hyesun Lee.

**Writing – original draft:** Hyesun Lee, Hyuk Sung Kwon.

**Writing – review & editing:** Hyesun Lee, Hyuk Sung Kwon.
